# Association of Hormone Therapy With Depression During Menopause in a Cohort of Danish Women

**DOI:** 10.1001/jamanetworkopen.2022.39491

**Published:** 2022-11-01

**Authors:** Marie K. Wium-Andersen, Terese S. H. Jørgensen, Anniken H. Halvorsen, Birgitte H. Hartsteen, Martin B. Jørgensen, Merete Osler

**Affiliations:** 1 Center for Clinical Research and Prevention, Bispebjerg and Frederiksberg Hospitals, Frederiksberg, Denmark; 2 Department of Public Health, University of Copenhagen, Copenhagen, Denmark; 3 Psychiatric Centre Copenhagen, Rigshospital, Copenhagen, Denmark

## Abstract

**Importance:**

During menopause, the levels of estrogen and progesterone decrease and 60% to 70% of women experience menopausal symptoms, including mood disturbances. The latter might be prevented by hormone therapy (HT), yet some studies have suggested that use of HT might be associated with increased risk of depression.

**Objective:**

To examine whether use of HT during menopause was associated with a subsequent diagnosis of depression.

**Design, Setting, and Participants:**

This nationwide register–based cohort and self-controlled case series study included all women in Denmark aged 45 years between January 1, 1995, through December 31, 2017 (n = 825 238), without prior oophorectomy, breast cancer, or cancer in reproductive organs. Follow-up was completed on December 31, 2018. The statistical analysis was performed from September 1, 2021, through May 31, 2022.

**Exposures:**

Redeemed prescriptions of different types of HT identified by the Anatomical Therapeutic Chemical classification system codes (G03C [estrogen] and G03F [estrogen combined with progestin]) in the Danish National Prescription Registry between 1995 and 2017. Type of administration was divided into systemic (oral or transdermal) and local (intravaginal or intrauterine).

**Main Outcomes and Measures:**

A hospital diagnosis of depression (*International Statistical Classification of Diseases and Related Health Problems, Tenth Revision*, codes F32-F33 and *International Classification of Diseases, Eighth Revision*, codes 296.09, 296.29, 298.0, and 300.49) between 1995 through 2018. Associations were examined in cohort and self-controlled case series analysis using Cox proportional hazards and fixed-effects Poisson regression models.

**Results:**

During follow-up from 45 years of age to a mean of 56.0 (range, 45.1-67.7) years, 189 821 women (23.0%) initiated systemically or locally administered HT and 13 069 (1.6%) were diagnosed with depression. Systemically administered HT was mainly initiated before 50 years of age and was associated with a higher risk of a subsequent depression diagnosis (hazard ratio [HR] for 48-50 years of age, 1.50 [95% CI, 1.24-1.81]). The risk was especially elevated the year after initiation of both treatment with estrogen alone (HR, 2.03 [95% CI, 1.21-3.41]) and estrogen combined with progestin (HR, 2.01 [95% CI,1.26-3.21]). Locally administered HT was initiated across all ages and was not associated with depression risk (HR, 1.15 [95% CI, 0.70-1.87]). It was, however, associated with a lower risk of depression when initiated after 54 years of age (HR for 54-60 years of age, 0.80 [95% CI, 0.70-0.91]). In self-controlled analysis, which efficiently accounts for time-invariant confounding, users of systemically administered HT had higher rates of depression in the years after initiation compared with the years before treatment (incidence rate ratio for 0-1 year after initiation, 1.66 [95% CI, 1.30-2.14]).

**Conclusions and Relevance:**

These findings suggest that systemically administered HT before and during menopause is associated with higher risk of depression, especially in the years immediately after initiation, whereas locally administered HT is associated with lower risk of depression for women 54 years or older.

## Key Points

**Question:**

Is hormonal therapy (HT) during menopause associated with risk of developing depression?

**Findings:**

In this cohort study including all Danish women aged 45 years followed up for a mean of 11 years, initiation of systemically administrated HT before 50 years of age was associated with higher risk of depression. Locally administered HT was associated with lower risk of depression for women older than 54 years.

**Meaning:**

These findings suggest that women undergoing menopause who initiate systemically administered HT should be aware of depression as a potential adverse effect, and locally administered HT should be recommended when needed.

## Teaser

This nationwide cohort study assesses the association of first-time use of specific types of hormone therapy with the risk of being diagnosed with depression among women in Denmark followed up from 45 years of age.

## Introduction

Depression is one of the leading causes of years lived with disability worldwide.^1^ Women are twice as likely as men to experience depression,^2^ suggesting that cyclic fluctuations in gonadal hormones—estrogen and progesterone—may contribute to this problem.^3,4,5^ During menopause, which typically occurs during the fifth decade of life, the levels of estrogen and progesterone decrease. In this period, 60% to 70% of women experience menopausal symptoms as well as mood and cognitive disturbances,^6,7^ including symptoms of depression.^8,9,10^

A few small randomized clinical trials (<200 participants)^11,12,13,14,15,16,17,18^ have shown that transdermal or oral estradiol plus micronized progesterone might prevent the development of depressive symptoms in women during menopause. In contrast, 2 larger randomized clinical trials^19,20^ have not supported a role of HT in the prevention of depression in postmenopausal women. Bothersome vulvovaginal symptoms may also influence quality of life, and a single experimental study has shown that vaginally administered estrogen may improve menopause-related quality of life,^21^ which in turn might lower the risk of depression. Cross-sectional studies have shown positive correlations between current use of hormone therapy (HT) and depressive symptoms in women around menopause^22,23,24,25,26^ or negative correlations in women older than 60 years, in whom HT seems associated with lower rates of depressive symptoms.^27^ The results from 7 longitudinal studies,^28,29,30,31,32,33,34^ in which the association between use of HT and changes in depressive symptoms during the years around menopause have been explored, have been inconclusive. No prior study seems to have assessed the association of different types of HT (locally vs systemically administered) with subsequent risk of clinically verified depression in a prospective cohort design, taking temporality between the use of HT and development of depression into account and including censoring due to mortality or loss to follow-up.

Although HT is widely used to manage menopausal symptoms,^35^ evidence of an effect of HT on the risk of developing depression is lacking. The aim of this study was to assess the association of first-time use of specific types of HT with the risk of being diagnosed with depression among women followed up from 45 years of age.

## Methods

### Population

This nationwide register–based cohort study included all women in the Danish Civil Registration System^36^ who were born between January 1, 1950, and December 31, 1972, and were living in Denmark on the day they turned 45 years of age (n = 920 817) between January 1, 1995, and December 31, 2017. The women were followed up in the Danish National Patient Registry, which was established in 1977.^37^ Women with an oophorectomy (n = 19 176), breast cancer (n = 6925), or cancer in reproductive organs before the age of 45 years (n = 2908) were excluded. eTable 1 in the Supplement provides the codes from *International Classification of Diseases, Eighth Revision* (*ICD-8*), and *International Statistical Classification of Diseases and Related Health Problems, Tenth Revision* (*ICD-10*), used for definitions. To reduce prevalent user bias, we further excluded women who were registered with HT use before age 45 years (n = 58 624). The study was approved by the regional data protection authority. All data were retrieved from administrative registers and informed consent was not required. This study followed the Strengthening the Reporting of Observational Studies in Epidemiology (STROBE) reporting guideline.

### Exposure

Exposure included all redeemed prescriptions of estrogen and progestin in the study period (1995-2017) using anatomical therapeutic codes G03C (estrogen) and G03F (estrogen and progestin in combination) from the Danish National Prescription Registry, which holds information on all prescribed and redeemed drugs sold at Danish pharmacies since 1995.^38^ We used information on the number of redeemed prescriptions, type of administration, and type of HT. Type of administration was divided into systemic (oral or transdermal) and local (intravaginal or intrauterine) use. Drugs with systemic administration were further divided into estrogen only, or estrogen-progestin in combination. Data used to calculate nationwide time trends on prescriptions of HT were obtained from MEDSTAT.^39^

### Outcome

Depression diagnoses were identified in the Danish Psychiatric Central Research Register and the Danish National Patient Registry and by a main diagnosis or subdiagnosis *ICD-10* code of F32 to F33 or *ICD-8* code of 296.09, 296.29, 298.0, and 300.49. The Danish Psychiatric Central Research Register contains information on all admissions to psychiatric hospitals or wards in Denmark from 1969 onward.^40^ From 1995, all outpatient treatments and information from emergency departments were also included. In case of repeated depressive episodes, hospital contacts with depression recorded within less than 365 days of each other were categorized as the same episode.^41^

### Covariates

We included several potential confounders assumed to be associated with both HT use and depression^34,42,43^ as covariates in the analyses. We used information on marital status from the Civil Registration System and extracted data on highest earned educational attainment from the Education Register in Statistics Denmark.^44^ From the Danish Medical Birth Registry,^45^ we retrieved information on parity and smoking during pregnancy. Data on previous use of hormonal contraceptives, fertility hormones, antidepressants, or sleep medication were obtained from the Danish National Prescription Registry. Finally, we included the comorbid conditions listed in eTable 1 in the Supplement.

### Statistical Analysis

Data were analyzed from September 1, 2021, to May 31, 2022. The associations between HT use and depression were analyzed using Cox proportional hazards regressions with age as the underlying timescale for calculating hazard ratios (HRs) and 95% CIs. All women were followed up from 45 years of age until depression diagnosis, emigration, death, cancer in mammae or reproductive organs, oophorectomy, or end of follow-up (December 31, 2018), which ever came first. Hormone therapy was entered as a time-dependent variable, implicating that women changed exposure status from nonexposed to exposed when HT was initiated. A total of 7946 women with ongoing depression (defined by a depression diagnosis or a redeemed antidepressant prescription within 1 year before 45 years of age or initiation of HT) were excluded from the specific exposure period because they were not at risk of developing the outcome. However, women who were excluded owing to depression within the 1 year before their 45th birthday were allowed to reenter at the time of initiating HT if they did not have depression at this point. We also explored a dose-response association of HT in women treated with HT. Because we focused on the risk associated with the lower range of the distribution, we examined whether redeeming 2 to 4 or 5 or more prescriptions compared with 1 prescription was associated with increased risk of depression using Cox proportional hazards regression with entry at first HT and number of HT prescriptions (2-4 and ≥5) as a time-dependent variable. To explore whether the risk varied with time since HT initiation, we specifically examined the HRs within the first year, for 1 to 5 years, and for more than 5 years after study entry (45 years of age or at HT initiation). Moreover, because studies suggest a beneficial effect of HT treatment on the risk of depression during menopause, but not after menopause, we also analyzed data in models with the follow-up time subdivided in 7 age bands around menopause (45-47, 48-50, 51-53, 54-56, 57-59, 60-62, and 63-68 years). In addition, women with prior depression may be more vulnerable to use of HT than those without previous depression. To explore whether depression history modified any association between HT and depression risk, we performed analyses stratified by previous depression (any previous diagnosis and/or use of antidepressant medication) and, if applicable, tested using likelihood ratio tests. Finally, we examined the influence of alterations made in national HT guidelines for use of systemic HT in 2002, which led to decline in use of systemic HT (eFigure 1 in the Supplement).^46^ In these analyses, HT users were divided according to time of exposure: HT use solely before January 1, 2003, or HT use continued or initiated after this date. All regression models were run with and without adjustment for covariates and stratified on birth cohort. The proportional hazards assumption was examined using Schoenfeld residuals.

To further account for time-invariant confounding, we used a self-controlled time series analysis^47,48^ that only included participants who initiated HT and experienced an outcome during follow-up. In these analyses, the potential association between HT and a depressive episode was investigated by comparing different time periods for each individual. In this analysis, time around the start of HT was divided into 6 study periods for each woman: (1) time when unexposed to HT (3 or 2 years before HT initiation); (2) a 1-year preexposure period up to and including the date of first prescription of HT; (3) the first year after HT initiation (initial exposed time); (4) the second year after HT initiation (late exposed time); and (5) the following year (consolidated exposed time) of follow-up. We used fixed-effects Poisson regression to calculate the incidence rates of depression episodes in the different study periods and generated incidence rate ratios with 95% CIs using the nontreatment period as reference. Two-sided *P* < .05 indicated statistical significance. Data were analyzed using STATA, version 17 (StataCorp LLC).

## Results

A total of 825 238 women who turned 45 years between 1995 and 2015 were included in the cohort. During follow-up (from 45 years of age to mean age of 56.0 [range, 45.1-67.7] years), a total of 189 821 women initiated HT (23.0%), and 13 069 (1.6%) were hospitalized for depression with an incidence of 17.3 (95% CI, 17.1-17.6) cases per 10 000 person-years.

### Characteristics of HT Users

Median age at first HT purchase was 55.0 (IQR, 49.0-56.0), and the median number of redeemed prescriptions of HT was 33 (IQR, 17-55). Most women redeemed 5 or more prescriptions (59.4%), and locally administered estrogen was the most common HT (65.8%). Fewer than one-fifth of the women (25 160 [17.3%]) shifted between the different types of HT (either between locally or systemically administered or between estrogen or combination therapy). Hormone therapy users were more often married (70.3% vs 63.3%) and had more than 1 childbirth (65.0% vs 60.2%), and fewer had used hormonal contraceptives (36.5% vs 54.0%) or fertility hormones (3.4% vs 6.1%) previously. Furthermore, HT users had fewer comorbidities, including prior depression (13.5% vs 20.6%) and diabetes (1.1% vs2.7%), and fewer reported smoking during pregnancy (8.2% vs 15.3%) (Table 1).

**Table 1.** Baseline Characteristics of Danish Women by Use of HT

Characteristic	Participant group, No. (%)^a^		
	All women (n = 825 238)	Non-HT users (n = 635 417)	HT users (n = 189 821)
Year of birth			
1950-1954	162 278 (19.7)	89 369 (14.1)	72 909 (38.4)
1955-1959	162 400 (19.7)	106 863 (16.8)	55 537 (29.3)
1960-1964	177 343 (21.5)	136 119 (21.4)	41 224 (21.7)
1965-1969	184 010 (22.3)	166 208 (26.2)	17 802 (9.4)
1970-1972	139 207 (16.9)	136 858 (21.5)	2349 (1.2)
Highest earned educational level^b^			
Low	190 682 (23.1)	142 588 (22.4)	48 094 (25.3)
Middle	369 155 (44.7)	289 634 (45.5)	79 521 (41.9)
High	252 013 (30.5)	192 706 (30.3)	59 307 (31.2)
Unknown	13 388 (1.6)	10 489 (1.7)	2899 (1.5)
Marital status			
Unmarried	152 787 (18.5)	127 132 (20.0)	25 655 (13.5)
Married	535 933 (64.9)	402 460 (63.3)	133 473 (70.3)
Divorced and widowed	136 518 (16.5)	105 825 (16.7)	30 693 (16.2)
No. of live or still births			
0	135 553 (16.5)	106 672 (16.8)	28 881 (15.2)
1	183 302 (22.2)	145 743 (22.9)	37 559 (19.8)
2-3	475 014 (57.5)	359 486 (56.5)	115 528 (60.9)
>3	31 369 (3.8)	23 516 (3.7)	7853 (4.1)
Prior use of hormonal contraceptives	413 000 (50.0)	343 411 (54.0)	69 231 (36.5)
Prior use of progestin	77 695 (9.4)	58 632 (9.2)	19 001 (10.0)
Prior use of fertility hormones	44 731 (5.4)	38 459 (6.1)	6361 (3.4)
Prior hysterectomy	8703 (1.1)	4742 (0.7)	4348 (2.3)
Prior depression	20 142 (2.4)	17 854 (2.8)	2288 (1.2)
Prior postpartum depression	5102 (0.6)	4732 (0.7)	370 (0.2)
Prior use of antidepressants	156 655 (19.0)	130 949 (20.6)	25 706 (13.5)
Prior use of sleeping medication	119 225 (14.4)	93 347 (14.7)	25 878 (13.6)
Comorbid diabetes	19 113 (2.3)	17 042 (2.7)	2071 (1.1)
Comorbid hypertension	11 128 (1.3)	9027 (1.4)	2101 (1.1)
Comorbid stroke	2321 (0.3)	2047 (0.3)	275 (0.1)
Comorbid heart disease	9370 (1.1)	7264 (1.1)	2106 (1.1)
Smoking habits in pregnancy^c^			
Smokers	112 927 (13.7)	97 462 (15.3)	15 465 (8.1)
Nonsmokers	287 272 (34.8)	248 589 (39.1)	38 683 (20.4)
Unknown^d^	425 039 (51.5)	289 366 (45.5)	135 673 (71.5)
Hormone therapy			
No. of prescriptions			
0	636 417 (77.0)	636 595 (100.0)	0
1	38 630 (4.7)	0	38 630 (20.4)
2-4	38 487 (4.7)	0	38 487 (20.3)
≥5	112 727 (13.6)	0	112 727 (59.4)
Route of administration			
Systemic (estrogen only)	14 808 (1.8)	0	14 808 (7.8)
Systemic (estrogen and progestin combined)	50 112 (6.1)	0	50 112 (26.4)
Local (estrogen)	124 904 (15.1)	0	124 901 (65.8)
Year of initiation			
1995-1999	12 136 (1.5)	0	12 136 (6.4)
2000-2004	31 630 (3.8)	0	31 630 (16.7)
2005-2009	40 668 (4.9)	0	40 668 (21.4)
2010-2014	54 052 (6.6)	0	54 050 (28.5)
2015-2018	51 338 (6.2)	0	51 337 (27.0)

Abbreviation: HT, hormone therapy.

^a^ Percentages have been rounded and may not total 100.

^b^ Highest earned educational level was categorized as primary school (low); high school or vocational education (middle); and higher educational level and higher advanced education (high).

^c^ For lack of a better measure, smoking habits in pregnancy was used as a proxy for smoking later in life.

^d^ Missing for births before 1996 and women not giving birth.

### Hormone Therapy and Depression

**Figure 1.**
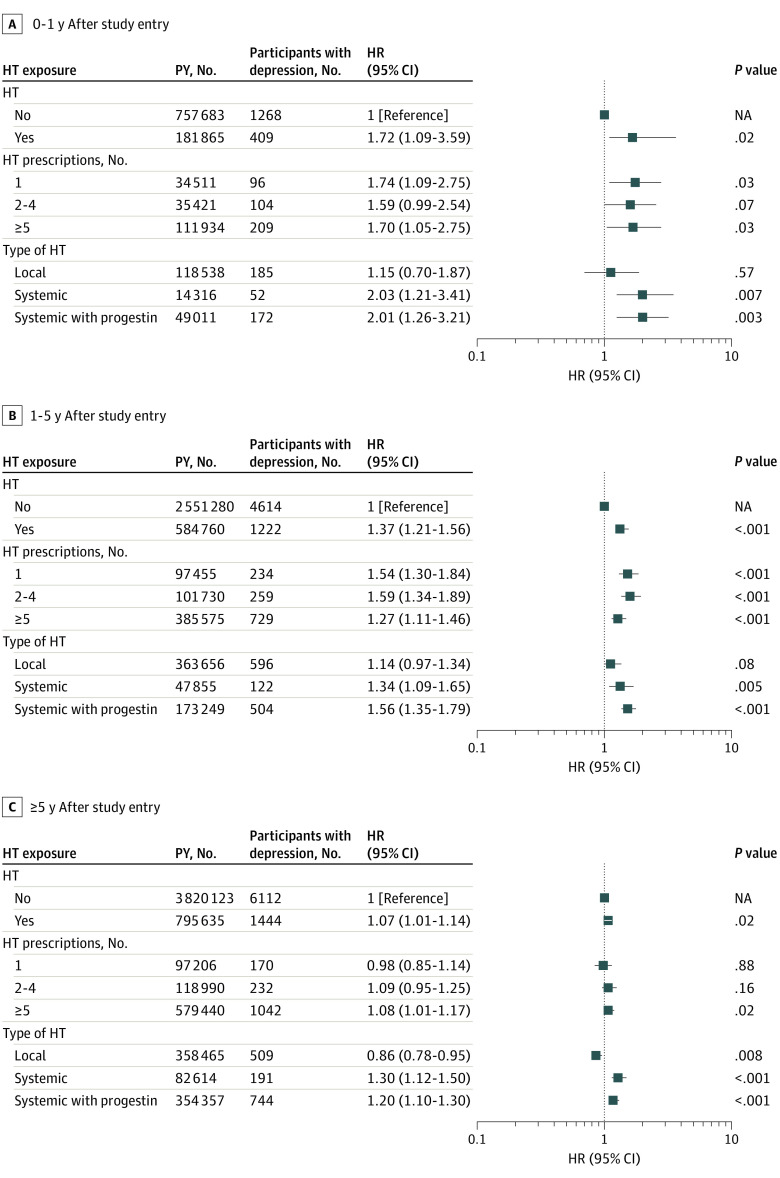
Associations Between Use of Hormone Therapy (HT) and Depression in Women Aged 45 Years by Follow-up All hazard ratios (HRs) were adjusted for educational level, marital status, number of still or live births, prior use of hormonal contraceptives, fertility hormones or progestin, prior hysterectomy, prior depression, postpartum depression, diabetes, heart disease, stroke, hypertension, smoking during pregnancy, and use of sleep medication. All analyses were stratified by birth year. NA indicates not applicable; PY, person-years.

**Figure 2.**
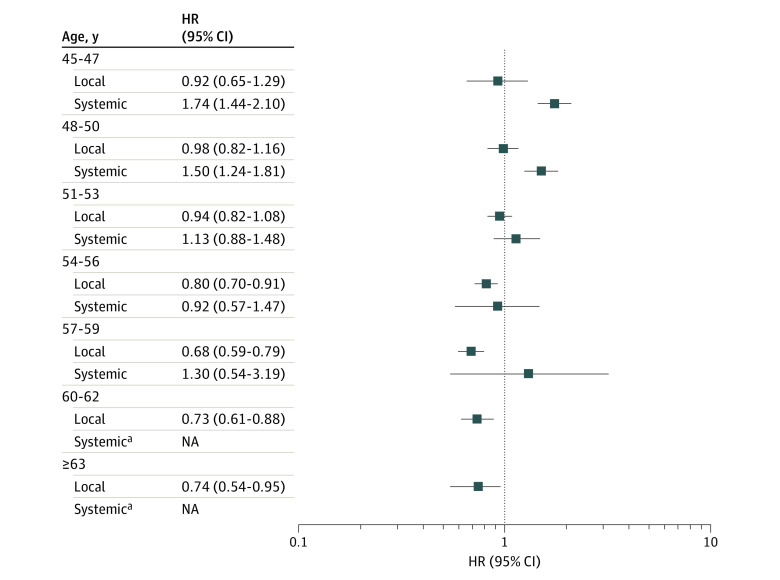
Adjusted Hazard Ratios (HRs) of Depression for Hormone Therapy by Age at Initiation All HRs were adjusted for educational level, marital status, number of still or live births, prior use of hormonal contraceptives, fertility hormones or progestin, prior hysterectomy, prior depression, postpartum depression, diabetes, heart disease, stroke, hypertension, smoking during pregnancy, and use of sleep medication and stratified by birth year. Error bars indicate 95% CIs. NA indicates not applicable. ^a^Indicates too few observations.

The crude incidence of depression was higher in women after initiation of HT than in nonusers (incidence, 19.6 [95% CI, 19.0-20.4] vs 16.8 [95% CI, 16.5-17.1] per 10 000 person-years). Compared with nonusers, users of HT experienced a higher risk of being diagnosed with depression. The risk was highest the first year after initiation (HR, 1.72 [95% CI, 1.09-3.59]) and declined gradually with time since initiation. The increased risk was independent of the number of redeemed HT prescriptions. The risk was especially elevated the year after initiation of both treatment with estrogen alone (HR, 2.03 [95% CI, 1.21-3.41]) and estrogen combined with progestin (HR, 2.01 [95% CI,1.26-3.21]) (Figure 1). Only systemically administered HT was associated with an increased risk of depression with no difference between estrogen alone or in combination with progestin. The route of administration varied with age at initiation. Before and during menopause, women more often started treatment with systemic HT, whereas locally administered estrogen also was initiated by women older than 56 years (eTable 2 in the Supplement). For systemically administered HT, the age-stratified analysis demonstrated a higher risk of depression among younger women (HR for 48-50 years of age, 1.50 [95% CI, 1.24-1.81]) and a lower risk of depression with greater age (HR for 51-53 years of age, 1.13 [95% CI, 0.88-1.48]). Conversely, locally administered HT was not associated with depression risk in women younger than 54 years (HR for age 48-50, 0.98 [95% CI, 0.82-1.16 ]) and was associated with a lower risk of depression for those aged 54 to 56 years (HR, 0.80 [95% CI, 0.70-0.91]) (Figure 2). When data were analyzed in relation to depression history, the estimates seemed to be slightly stronger in women with a previous depressive episode (Table 2), but the risk estimates were imprecise and the likelihood ratio test had *P* = .05. Use of systemically administered HT decreased after 2002 (eFigure 1 in the Supplement) and with increasing birth year (eFigure 2 in the Supplement), but an association between systemically administered HT and depression risk was seen both for HT prescribed before or after 2003 (eTable 3 in the Supplement).

**Table 2.** Adjusted Hazard Ratio of First Diagnosis of Depression After Initiation of HT in Women With or Without Previous Depression Followed Up From 45 Years of Age^a^

Participant group	Time after initiation of HT, y								
	0-1			1-5			5-23		
	Person-years	No. of cases	HR (95% CI)	Person-years	No cases	HR (95% CI)	Person-years	No cases	HR (95% CI)
Without previous depression (n = 649 279)									
No HT	649 195	534	1 [Reference]	2 221 760	2587	1 [Reference]	3 503 133	4434	1 [Reference]
HT	139 539	135	1.48 (0.66-3.34)	457 989	519	1.28 (1.05-1.55)	668 610	923	1.18 (1.09-1.27)
Locally administered HT	90 743	61	1.04 (0.43-2.40)	283 858	234	0.95 (0.74-1.21)	289 047	294	0.89 (0.79-1.01)
Systemically administered HT	48 796	74	1.67 (0.73-3.76)	264 031	285	1.44 (1.17-1.75)	370 563	629	1.38 (1.26-1.51)
With previous depression (n = 175 959)									
No HT	108 488	734	1 [Reference]	329 521	2037	1 [Reference]	316 989	1678	1 [Reference]
HT	42 326	374	1.84 (1.05-3.19)	126 771	703	1.43 (1.20-1.71)	127 026	521	0.90 (0.79-1.01)
Locally administered HT	27 795	124	1.18 (0.65-2.13)	79 798	362	1.28 (1.04-1.57)	60 418	215	0.81 (0.69-0.95)
Systemically administered HT	14 532	150	2.21 (1.26-3.86)	36 982	341	1.53 (1.27-1.84)	66 608	306	0.96 (0.84-1.11)

Abbreviations: HR, hazard ratio; HT, hormone therapy.

^a^ Adjusted for educational level, marital status, number of live or still births, prior use of hormonal contraceptives, fertility hormones or progestin, use of sleep medication, prior hysterectomy, diabetes, heart disease, stroke, hypertension, and smoking during pregnancy and stratified on birth year.

**Figure 3.**
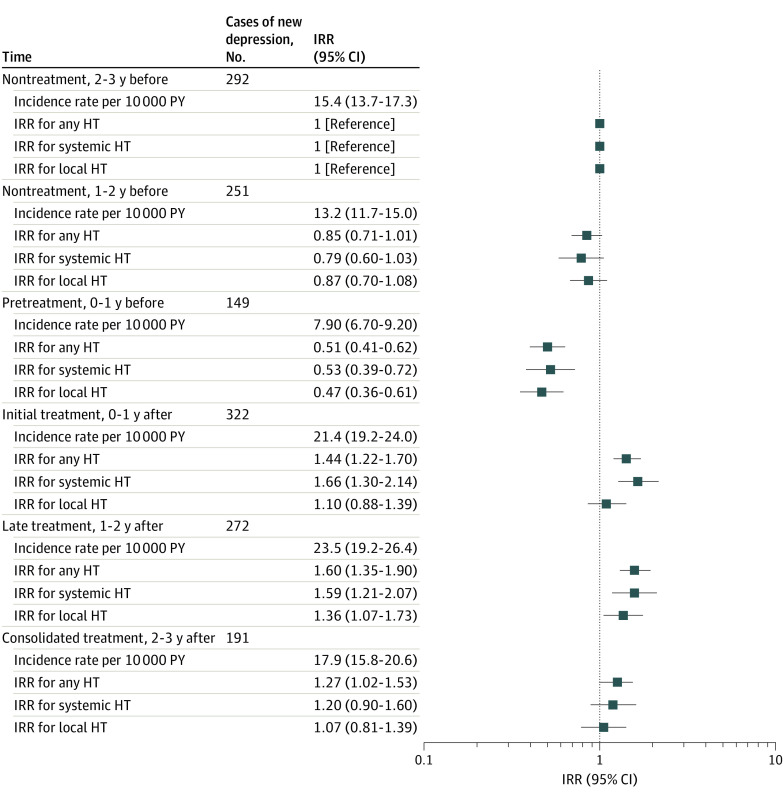
Incidence Rates and Incidence Rate Ratios (IRRs) of Depression in Different Study Periods Before and After Initiation in 189 821 Women 45 Years or Older Starting Hormone Therapy (HT) Error bars indicate 95% CIs. PY indicates person-years.

In the self-controlled analysis comparing different time periods related to HT initiation, the incidence rate of depression was higher after HT initiation than before (within 1 year before treatment, 7.9 [95% CI, 6.7-9.2] per 10 000 person-years; 1-2 years after treatment, 23.5 [95% CI, 19.2-26.4] per 10 000 person-years). When compared with the nontreatment period (2-3 years before HT initiation), the incidence rate ratios were higher in the 3 treatment periods (0-1 year after initiation, 1.44 [95% CI, 1.22-1.70]; 1-2 years after initiation, 1.60 [95% CI, 1.35-1.90]) and significantly lower in the pretreatment period (1-0 year before initiation, 0.51 [95% CI, 0.41-0.62]) (Figure 3).

## Discussion

In this nationwide cohort study, initiation of systemically administered HT was associated with a subsequent diagnosis of depression. The risk was highest in women aged 45 to 50 years and during the first years after starting HT. Locally administered HT was not associated with risk of depression in women younger than 54 years and was associated with a lower risk for those 54 years or older.

A major strength of our study was the large nonselective inclusion of all women aged 45 years living in Denmark, who were followed up for as long as 22 years with almost no loss to follow-up. The information on use of HT was obtained from nationwide registers, with HT only being available by prescription in Denmark, thereby limiting information bias. Because prescription medication in Denmark is not free, we assume that most women who purchase HT are likely to start treatment. Data for HT were continuously updated and included as a time-varying exposure variable to limit immortal time bias. We included several potential confounders in the analysis, and to address confounding more efficiently, we also used a self-controlled analysis adjusting for all time-invariant confounders.

### Comparison With Previous Studies

Our study seems to be the first nationwide observational study that has followed up women who initiate HT for clinically verified depression in time-to-event analyses. However, a recent cohort study^49^ followed up 291 709 US women older than 50 years for 4.5 years and found that HT was associated with a 2 times higher risk of suicide, which may be an indicator of severe depression. A review from 2015 including 19 experimental studies on the effect of systemically administered HT for developing depressive symptoms in women without depression^17^ provided no support for an effect of oral or transdermal estrogen alone or combined with progestin on mood in nondepressed women. However, only 5 of the studies (<200 women) included women younger than 60 years at baseline, and these studies provided inconsistent results. Since 2015, 2 experimental studies showed that transdermal or oral estrogen were more effective than placebo in preventing depressive symptoms in nondepressed women around menopause.^14,50^ We also identified 18 original observational studies, assessing the association between use of HT and depressive symptoms, which are described in detail in eTables 4 and 5 in the Supplement. One of the 11 cross-sectional studies^27^ demonstrated correlations between estrogen use and lower frequency of depressive symptoms. However, in the 2 largest recent studies,^24,26^ use of HT correlated with worse depression scores among 6000 and 13 000 perimenopausal and postmenopausal women, respectively. Among 7 longitudinal cohort studies,^28,29,30,31,32,33,34^ HT use was associated with depression scores at baseline or follow-up in two^31,32^ (eTable 5 in the Supplement). All longitudinal studies were based on self-reported information on HT use and depressive symptoms and had high rates of nonresponse both at baseline (15%-63%) and follow-up (9%-50%). Moreover, because hormone preparation was often not indicated, it is difficult to evaluate the impact of type of HT or route of administration on depression risk from previous studies. The biological mechanisms linking HT and mood disturbances have suggested that estradiol only has beneficial mood and neuroprotective effects if administered proximate to the cessation of ovarian activity, whereas this may be opposite at later stages.^11^

### Limitations

This study has some limitations. Although we used different approaches to address potential confounding, there is still risk of unmeasured confounding, especially owing to the lack of information on menopausal status and symptoms, including impaired mood, which might be important confounders. Menopause increases the risk of both HT and depression,^6^ which could explain some of the higher risk of depression in HT users. The self-controlled analysis showed that the rate of depression was lower especially before HT prescription, which might indicate that clinicians in general practice are less likely to prescribe HT to women at risk of depression. This opposite selection implies a potential underestimation of the HR. On the other hand, if prescribing physicians are more observant of the onset of depressive symptoms among patients to whom they have prescribed HT, this could introduce detection bias. However, such bias may not explain the increased risk for a first depression diagnosis at a psychiatric hospital because these diagnoses reflect the more severe depressive disorders that will most likely be evident regardless of clinical attention. Another possible explanation for the lower rate of depression before HT prescription is that depression in menopause may be interpreted as menopausal symptoms and treated as such. If the misinterpreted depressive symptoms worsen owing to insufficient treatment, an overestimation of the HT would result. However, some experimental studies have suggested an antidepressant effect of HT in women with ongoing depression,^8,17,43^ and this could counter an overestimation of the HR. To account for such bias, we included a pretreatment period (Figure 3) just before exposure to HT, anticipating that the chance of starting treatment with HT could temporally be altered by having depression or depressive symptoms. Our outcome data were based on hospital diagnoses of depression and may not include women with milder depression episodes treated in primary care. This might have led to an underestimation of the HRs if the misclassification was random. We did consider using purchases of antidepressants as a secondary outcome but desisted from this approach owing to risk of bias. Thus, antidepressants might be prescribed for other menopausal symptoms such as pain, sleeping disorder, and anxiety, which could also be associated with initiation of HT. Consequently, we might overestimate any risk of depression associated with HT. We found that the risk of this potential bias may be much smaller for depression diagnosis.

Our findings suggest that around menopause, women may be more sensitive to the influence of HT on mood than at later ages. This finding could be influenced by attrition of susceptibility to HT, but we could not calculate precise risk estimates for use of systemic HT in menopausal women older than 54 years because less than 1% initiated treatment with systemic HT after 54 years of age. Finally, we cannot exclude that the lower risk among women using locally administered HT compared with the corresponding systemically administered HT may be explained by a lower contribution of locally acting HT to the systemic circulation or that menopausal symptoms, including depression, are more likely to be treated with systemic HT.

## Conclusions

The findings of this cohort study suggest that during menopause, use of systemically administered HT is associated with risk of a subsequent diagnosis of depression among women living in Denmark. Locally administered HT is not associated with depression risk and is even associated with a possible lower risk for women older than 54 years, and may therefore be recommended when appropriate.

## Notes

Supplement. **eFigure 1.** Number per 1000 Women Aged 45-64 Years in Denmark Who Purchased Hormone Therapy, 1999-2019 **eFigure 2.** Proportion of Women Who Had Purchased Hormone Therapy (HT) Before Age 55 Years in Relation to Year of Birth **eTable 1.** *International Classification of Disease* (*ICD*) and Anatomical Therapeutical Chemical (ATC) Codes Used for Definition of Comorbid Conditions or Medication **eTable 2.** Percentage of Users of Hormone Therapy (HT) and Multiple Adjusted Hazard Ratio (HR) and 95% CIs of Depression for HT by Age at Initiation **eTable 3.** Adjusted Hazard Ratio of First Diagnosis of Depression After Initiation of Hormone Therapy (HT) in Women Before and After January 1, 2003 **eTable 4.** Overview of Cross-sectional Studies on Hormone Therapy (HT) and Depression **eTable 5.** Overview of Longitudinal Studies on Hormone Therapy (HT) and Depression
eReferences
