# Supplementary material for: Association of Hormone Therapy With Depression During Menopause in a Cohort of Danish Women
Source: JAMA Netw Open. 2022 Nov 1;5(11):e2239491. doi: 10.1001/jamanetworkopen.2022.39491 (PMC9627415; doi:10.1001/jamanetworkopen.2022.39491)
Supplement: Supplement. — eFigure 1. Number per 1000 Women Aged 45-64 Years in Denmark Who Purchased Hormone Therapy, 1999-2019 eFigure 2. Proportion of Women Who Had Purchased Hormone Therapy (HT) Before Age 55 Years in Relation to Year of Birth eTable 1. International Classification of Disease (ICD) and Anatomical Therapeutical Chemical (ATC) Codes Used for Definition of Comorbid Conditions or Medication eTable 2. Percentage of Users of Hormone Therapy (HT) and Multiple Adjusted Hazard Ratio (HR) and 95% CIs of Depression for HT by Age at Initiation eTable 3. Adjusted Hazard Ratio of First Diagnosis of Depression After Initiation of Hormone Therapy (HT) in Women Before and After January 1, 2003 eTable 4. Overview of Cross-sectional Studies on Hormone Therapy (HT) and Depression eTable 5. Overview of Longitudinal Studies on Hormone Therapy (HT) and Depression eReferences [file jamanetwopen-e2239491-s001.pdf]

Supplementary Online Content

Wium-Andersen MK, Jørgensen TSH, Halvorsen AH, Hartsteen BH, Jørgensen MB, Osler M. Association of hormone therapy with depression during menopause in a cohort of Danish women. *JAMA Netw Open*. 2022;5(11):e2239491. doi:10.1001/jamanetworkopen.2022.39491

eFigure 1. Number per 1000 Women Aged 45-64 Years in Denmark Who Purchased Hormone Therapy, 1999-2019
eFigure 2. Proportion of Women Who Had Purchased Hormone Therapy (HT) Before Age 55 Years in Relation to Year of Birth
eTable 1. *International Classification of Disease* (*ICD*) and Anatomical Therapeutical Chemical (ATC) Codes Used for Definition of Comorbid Conditions or Medication
eTable 2. Percentage of Users of Hormone Therapy (HT) and Multiple Adjusted Hazard Ratio (HR) and 95% CIs of Depression for HT by Age at Initiation
eTable 3. Adjusted Hazard Ratio of First Diagnosis of Depression After Initiation of Hormone Therapy (HT) in Women Before and After January 1, 2003
eTable 4. Overview of Cross-sectional Studies on Hormone Therapy (HT) and Depression
eTable 5. Overview of Longitudinal Studies on Hormone Therapy (HT) and Depression
eReferences

This supplementary material has been provided by the authors to give readers additional information about their work.

© 2022 Wium-Andersen MK et al. *JAMA Network Open*.

eFigure 1. Number per 1000 Women Aged 45-64 Years in Denmark Who Purchased Hormone Therapy, 1999-2019

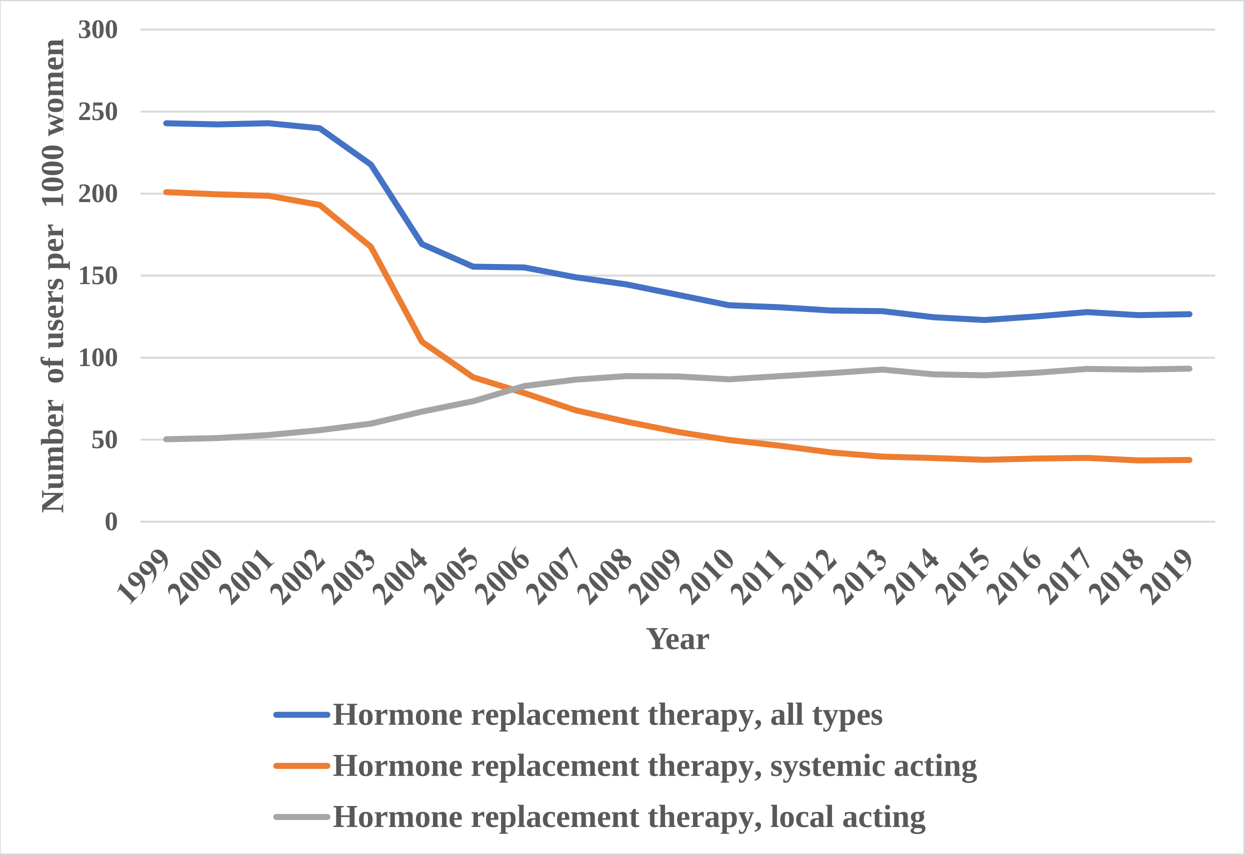

Ref: Own calculations based on data from www.medstat.dk

© 2022 Wium-Andersen MK et al. *JAMA Network Open*.

eFigure 2. Proportion of Women Who Had Purchased Hormone Therapy (HT) Before Age 55 Years in Relation to Year of Birth

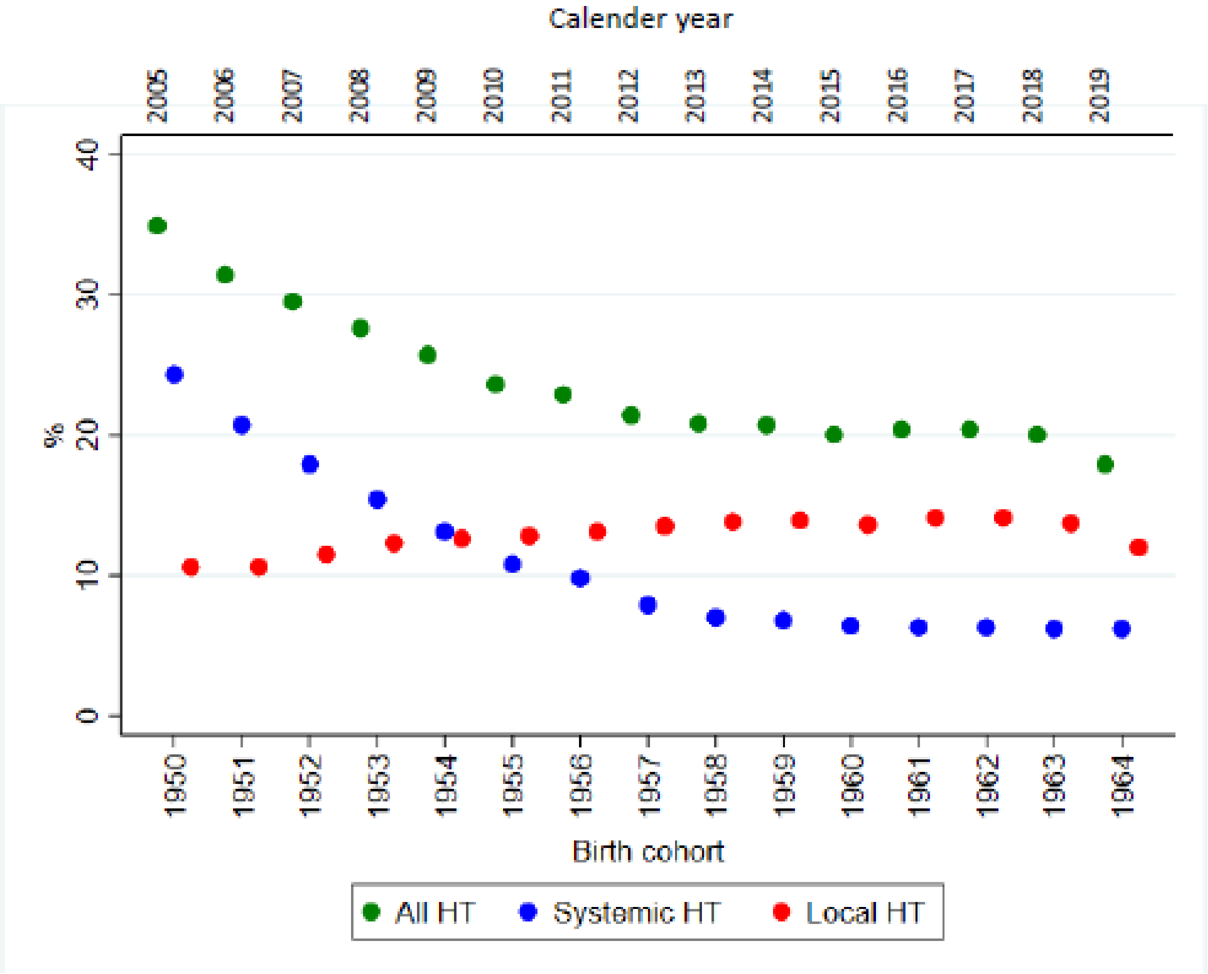

© 2022 Wium-Andersen MK et al. *JAMA Network Open*.

eTable 1. *International Classification of Disease* (*ICD*) and Anatomical Therapeutical Chemical (ATC) Codes Used for Definition of Comorbid Conditions or Medication

**Excluding diseases and surgical procedures**		
	**ICD-8 code**	**ICD-10 code**
Mamma cancer	17400, 17401, 17402, 17408, 17409	DC50, DC500, DC500A, DC500B, DC500C, DC500D, DC501, DC502, DC503, DC504, DC505, DC506, DC508, DC509
Cancer reproductive organs	18000, 18001, 18002, 18003, 18008, 18009, 18199, 18200, 18201, 18202, 18203, 18208, 18209, 18299, 18300, 18301, 18302, 18303, 18308, 18309, 18319, 18399, 18400, 18401, 18409, 18419, 18489, 18499	DC53, DC539, DC539M, DC539X, DC54, DC549, DC549M, DC549X, DC55, DC559, DC56, DC569, DC569A, DC569M, DC569X, DC57, DC570, DC570M, DC571, DC571M, DC572, DC572M, DC573, DC573M, DC574, DC574M, DC577, DC577M, DC578, DC578M, DC579, DC579M, DC579X
Hysterectomy	60920, 60940, 61000, 61020, 61040, 61050, 61100	KLCD30, KLCD31, KLCD40
Oophorectomy	60100, 60101, 60120, 60121, 60300, 60320	KLAF10, KLAF10A, KLAF11, KLAE10, KLAE10A, KLAE11, KLAE20, KLAE20A, KLAE21
**Comorbid conditions**		
Heart diseases	410-414	I20-I25
Stroke	431+433-434,435	I60-I69, G45
Hypertension	400-404	I10-I15
Diabetes	250	E10-E14
Post-partum depression	29609,29629,29800,30049 within the first year after delivery registered in the medical birth registry	F32, F33 within the first year after delivery registered in the medical birth registry
Hysterectomy	60920, 60940, 61000, 61020, 61040, 61050, 61100	KLCD30, KLCD31, KLCD40
**Co-medication**	**ATC codes**	
Hormone contraceptives	G03A, G02BA, G02BB	
Gestagenes	G03DA, G03DC	
Fertility hormones	G03G	
Antidepressants	N06A	
Sleep medication	N05CD, N05CF, NO5CH	

© 2022 Wium-Andersen MK et al. *JAMA Network Open*.

eTable 2. Percentage of Users of Hormone Therapy (HT) and Multiple Adjusted Hazard Ratio (HR) and 95% CIs of Depression for HT by Age at Initiation

Age	HT	Users %	Multiple adjusted			
			HR	lowerCI	higherCI	p,value
45-47	Local	20.0	0.92	0.65	1.29	0.632
	Systemic	45.0	1.74	1.44	2.1	<0.001
48-50	Local	31.7	0.98	0.82	1.16	0.769
	Systemic	41.6	1.5	1.24	1.81	<0.001
51-53	Local	16.3	0.94	0.82	1.08	0.456
	Systemic	9.4	1.13	0.88	1.48	0.071
54-56	Local	15.1	0.8	0.7	0.91	0.025
	Systemic	3.3	0.92	0.57	1.47	0.125
57-59	Local	9.2	0.68	0.59	0.79	0.087
	Systemic	0.6	1.3	0.54	3.19	0.231
60-62	Local	4.9	0.73	0.61	0.88	0.002
	Systemic	-	-	-	-	
63+	Local	.7	0.74	0.54	0.95	0.042
	Systemic	-	-	-	-	
Loglikelihood-ratio test						0.035

-too few observations

© 2022 Wium-Andersen MK et al. *JAMA Network Open*.

eTable 3. Adjusted Hazard Ratio of First Diagnosis of Depression After Initiation of Hormone Therapy (HT) in Women Before and After January 1, 2003

	**Person-years**	**No cases**	**Unadjusted Hazard ratio (95%CI)**	***P* value**	**Multiple adjusted Hazard ratio (95%CI)**	***P* value**
	**1995-2002** (n=174,482)					
No hormone therapy	917,924	1,395	1		1	
Hormone therapy	84,111	240	1.88 (1.62,2.17)	<0.001	1.27 (1.09-1.48)	0.002
Local administered hormone therapy	18,011	33	1.20 (0.85,1.71)	0.287	0.84 (0.91-1.20)	0.364
Systemic administered hormone therapy	66,100	207	2.06 (1.77,2.40)	<0.001	1.38 (1.18-1.62)	<0.001
	**2003-2018** (n=772,025)					
No hormone therapy	6,211,162	10,559	1		1	
Hormone therapy	1,478,149	2,835	1.32 (1.26,1.38)	<0.001	0.98 (0.93-1.02)	0.421
Local administered hormone therapy	655,501	1,257	1.06 (0.99,1.12)	0.062	0.79 (0.74-0.84)	<0.001
Systemic administered hormone therapy	822,648	1,578	1.64 (1.55,1.74)	<0.001	1.19 (1.13-1.26)	<0.001
* adjusted for education, marital status, number of deliveries, prior use of hormonal contraceptives, fertility hormones or progestin, prior hysterectomy, prior depression, post-partum depression, diabetes, heart disease, stroke, hypertension, smoking during pregnancy and stratified on birth year.						

© 2022 Wium-Andersen MK et al. *JAMA Network Open*.

eTable 4. Overview of Cross-sectional Studies on Hormone Therapy (HT) and Depression

Author year	Population	Age	Sample N	Exposure	Outcome	Adjustments	Results
Palinkas [1] 1992 USA	Middle- and upper-class women	50-89	1190	Current use of HT (25%)	Becks depression inventory (BDI)	Age	Current HT use did not correlate with depression score in all women. Current HT use correlated with higher depression score in women aged 50-59 years
Collins [2] 1994 Sweden	Population based selection of women	48	1324	Current use of HT (7.5%)	Depressive symptoms I the Menopause symptom inventory	No	Current users of HT had a higher rate of impaired mood
Whooley[3] 2000 USA	Population based selection of women	>71	6,602	Current (6.3%) past (7.2%) use of HT	Geriatric Depression Scale short from	Living alone, oophorectoomi, smoking, exercise, social network, self-perceived health, cognitive function and antidepressant use.	Current HT use correlated with lower depression score
Maartens [4] 2000 Netherlands	Population based selection of women	47-54	3,029	Current use of HT (10%)	Edinburg depression scale	Age, body mass index, parity, education, employment, marital status and smoking.	Current use correlated with higher depression score
Boosworth [5] 2000 USA	Population based selection of women	45-54	581	Ever (40.7%) Current (26.7) use of HT	Abbreviated CES-D	Menopausal status, age, marital status, race, income smoking, exercise	Current use of estrogen+progestrone associated with higher odds of depressive symptoms
Genazzani[6] 2002 Italy	Women visiting a menopause center	>45	2169	Current users of HT (n=1093) And a matched sample of non-users (n=1067)	Women health questionnaire depression subscale	Age, education, marital status, duration of menopause, chronic disease	Current use of HT was not correlated with depression
Lim [7] 2006 Korea	Volunteer visitors to a wellness clinic	40-60	135	Current use of HT (48%)	Zung self-rating depression scale	No	HT user correlated with lower depression score
Amore[8] 2007 Italy	Population based selection of women	45-55	1344	Current use of HT (11%)	Womens health questionnaire	Age, life events, place of residence, number of children, employment and marital status	Current use of HT was not correlated with depression
Toffol [9] 2013 Finland	Population based selection of women	40-74	6,760	Current use of any type of HT (28%)	BDI+ Self-reported depression diagnosis	Age, marital status, education, employment, psychiatric diagnosis, hysterectomy	Current use of HT correlated with higher BDI and recent depression diagnosis
Lee [10] 2016 Korea	Population based selection of women	Mean age 56	2,286	Current use of HT	Mental health survey	No	Current HT use correlated with higher depression score
Shea [11] 2020 Canada	Population based selection of women	45-64	13,216	Current use of HT (10%)	Center for epidemiologic studies short depression scale (CES-D)	Age, education, income, marital status, body mass index, alcohol use, social support and participation	Current use of HT associated with higher odds of depressive symptoms

© 2022 Wium-Andersen MK et al. *JAMA Network Open*.

eTable 5. Overview of Longitudinal Studies on Hormone Therapy (HT) and Depression

**Author year**	**Population**	**Baseline age**	**Sample N**	**Follow-up**	**Exposure**	**Outcome**	**Adjustments**	**Results**
Matthews[12] 1990 USA	Population based selection of women	42-50	202 Nb=40% Bf=?	3 years	HT use at follow-up (165)	Becks depression inventory (BDI)	Age, race, marital status, education, baseline health status	HT use was not associated with BDI at baseline or follow-up
Avis [13] 1994 USA	Population based selection of women	45-55	2565 Nb=23% Bf=9%	5 years	Ever use of HT (5.1%)	Center for epidemiologic studies short depression scale (CES-D)	Previous depression, menopausal symptoms	HT use was not associated with CES-D baseline or follow-up
Hardy[14] 2002 UK	Population based selection of women	47	1572 Nb=15% Bf=26%	5 years	HT use at baseline (10%) and/ or at follow-up (23%)	Psychological symptom score	Age, menopausal status, menopausal symptoms	HRT use at baseline or follow-up was associated with more psychological symptoms.
Cohen[15] 2006 USA	Women with no history of depression	36-45	460 Nb=27% Bf=30%	6 years	HT use at baseline	CES-D	Age and life experiences	Perimenopausal HT users had same rate of depression as non-users
Hess [16] 2008 USA	Population based selection of women	42-52	3,105 Nb=15% Bf=26%	6 years	HT use during follow-up (26%)	Health-related quality of life-vitality score	Age, ethnicity, education, comorbid condition	HT use during follow-up was not associated with subsequent vitality score
Scali[17] 2010 France	Population based selection of women	>65	4,069 Nb=63% Bf=30%	4 years (2 times with 2 years interval)	Current (14.7%) and past (19.9%) HT use at baseline and HT use at follow-up (17.9%)	CES-D	Age. Education, marital status, age at menopause, comorbidity, insomnia, disability and previous depression	HT use during follow-up was not associated with development of depressive symptoms
Hickey[18] 2016 Australia	Population based selection of women	45-50	5,895 Nb=47% Bf=30%	5 times with 3 years interval (15 years)	HT use at baseline (11.8)	CES-D	Age, occupation, smoking, alcohol use, body, mass index, exercise, menopausal symptoms, previous depression	Starting or stopping HRT were associated with higher depression scores during follow-up
Nb=percent non-responders at baseline; Nf=percent non-responders at last follow-up								

© 2022 Wium-Andersen MK et al. *JAMA Network Open*.

eReferences

1. Palinkas LA, Barrett-Connor E. Estrogen use and depressive symptoms in postmenopausal women. *Obstet Gynecol*. 1992;80(1):30-36.

2. Collins A, Landgren BM. Reproductive health, use of estrogen and experience of symptoms in perimenopausal women: a population-based study. *Maturitas*. 1994;20(2-3):101-111. doi:10.1016/0378-5122(94)90005-1

3. Whooley MA, Grady D, Cauley JA. Postmenopausal estrogen therapy and depressive symptoms in older women. *J Gen Intern Med*. 2000;15(8):535-541. doi:10.1046/j.1525-1497.2000.04029.x

4. Maartens LW, Leusink GL, Knottnerus JA, Pop VJ. Hormonal substitution during menopause: what are we treating? *Maturitas*. 2000;34(2):113-118. doi:10.1016/s0378-5122(99)00093-6

5. Bosworth HB, Bastian LA, Kuchibhatla MN, et al. Depressive symptoms, menopausal status, and climacteric symptoms in women at midlife. *Psychosom Med*. 2001;63(4):603-608. doi:10.1097/00006842-200107000-00013

6. Genazzani AR, Nicolucci A, Campagnoli C, et al. Assessment of the QoL in Italian menopausal women: comparison between HRT users and non-users. *Maturitas*. 2002;42(4):267-280. doi:10.1016/s0378-5122(02)00067-1

7. Lim HJ, Cho HJ, Lee MS. Pilot study of hormone replacement therapy and menopausal symptoms, depression, and quality of life in Korean climacteric women. *Psychol Rep*. 2006;98(2):374-378. doi:10.2466/pr0.98.2.374-378

8. Amore M, Di Donato P, Berti A, et al. Sexual and psychological symptoms in the climacteric years. *Maturitas*. 2007;56(3):303-311. doi:10.1016/j.maturitas.2006.09.006

9. Toffol E, Heikinheimo O, Partonen T. Associations between psychological well-being, mental health, and hormone therapy in perimenopausal and postmenopausal women: results of two population-based studies. *Menopause N Y N*. 2013;20(6):667-676. doi:10.1097/gme.0b013e318278eec1

10. Lee JY, Park YK, Cho KH, et al. Suicidal ideation among postmenopausal women on hormone replacement therapy: The Korean National Health and Nutrition Examination Survey (KNHANES V) from 2010 to 2012. *J Affect Disord*. 2016;189:214-219. doi:10.1016/j.jad.2015.09.068

11. Shea AK, Sohel N, Gilsing A, Mayhew AJ, Griffith LE, Raina P. Depression, hormone therapy, and the menopausal transition among women aged 45 to 64 years using Canadian Longitudinal Study on aging baseline data. *Menopause N Y N*. 2020;27(7):763-770. doi:10.1097/GME.0000000000001540

12. Matthews KA, Wing RR, Kuller LH, et al. Influences of natural menopause on psychological characteristics and symptoms of middle-aged healthy women. *J Consult Clin Psychol*. 1990;58(3):345-351. doi:10.1037//0022-006x.58.3.345

© 2022 Wium-Andersen MK et al. *JAMA Network Open*.

13. Avis NE, Brambilla D, McKinlay SM, Vass K. A longitudinal analysis of the association between menopause and depression. Results from the Massachusetts Women's Health Study. *Ann Epidemiol*. 1994;4(3):214-220. doi:10.1016/1047-2797(94)90099-x

14. Hardy R, Kuh D. Change in psychological and vasomotor symptom reporting during the menopause. *Soc Sci Med*. 2002;55(11):1975-1988. doi:10.1016/S0277-9536(01)00326-4

15. Cohen LS, Soares CN, Vitonis AF, Otto MW, Harlow BL. Risk for New Onset of Depression During the Menopausal Transition: The Harvard Study of Moods and Cycles. *Arch Gen Psychiatry*. 2006;63(4):385-390. doi:10.1001/archpsyc.63.4.385

16. Hess R, Colvin A, Avis NE, et al. The impact of hormone therapy on health-related quality of life: longitudinal results from the Study of Women's Health Across the Nation. *Menopause N Y N*. 2008;15(3):422-428. doi:10.1097/gme.0b013e31814faf2b

17. Scali J, Ryan J, Carrière I, et al. A prospective study of hormone therapy and depression in community-dwelling elderly women: the Three City Study. *J Clin Psychiatry*. 2010;71(12):1673-1679. doi:10.4088/JCP.09m05188blu

18. Hickey M, Schoenaker DAJM, Joffe H, Mishra GD. Depressive symptoms across the menopause transition: findings from a large population-based cohort study. *Menopause N Y N*. 2016;23(12):1287-1293. doi:10.1097/GME.0000000000000712

19. Toffol E, Heikinheimo O, Partonen T. Hormone therapy and mood in perimenopausal and postmenopausal women: a narrative review. *Menopause N Y N*. 2015;22(5):564-578. doi:10.1097/GME.0000000000000323

© 2022 Wium-Andersen MK et al. *JAMA Network Open*.
